# Placental mRNA and miRNA dynamics associated with lipid metabolism pathways in pregnancies affected by obesity

**DOI:** 10.3389/fendo.2025.1736033

**Published:** 2026-01-28

**Authors:** Ruggero Spadafora, Jiayi Zhang, N. R. Nirmala, Xue Li, Perrie O’Tierney-Ginn

**Affiliations:** 1 Woman, Mother + Baby Research Institute, Tufts Medical Center, Boston, MA, United States; 2 Department of Pediatrics, Division of Neonatology Tufts Medical Center, Boston, MA, United States; 3 Department of Data Analytics, Tufts University, Boston, MA, United States; 4 Institute for Clinical Research and Health Policy Studies (ICRHPS) Tufts Medical Center, Boston, MA, United States; 5 Tufts Technology Services Research Technology, Tufts University, Medford, MA, United States

**Keywords:** free fatty acids, maternal obesity, microRNA, placenta, transcriptome

## Abstract

**Introduction:**

Maternal obesity (pregravid body mass index >30 kg/m^2^), which has reached epidemic levels in the US, increases the incidence of cardiovascular disease and all cause premature death in the offspring. The placenta modulates fetal access to lipids and other nutrients and is considered a key player in fetal growth and maturation. However, the complex interplay between dysregulated metabolism in mothers with obesity and placental pathways mediating impacts on fetal development that predispose offspring to morbidities later in life, is poorly understood.

**Methods:**

We used unbiased *Whole Genome Correlation Network Analysis* (WGCNA) in 39 full-term unlabored placentas from mothers affected by obesity to explore relationships between coding and non-coding placental transcripts with maternal and fetal metabolic variables.

**Results:**

We identified positive correlations between members of the Rho network, a key inflammation regulator, with maternal leptin and cord blood free fatty acids (cbFFA). Furthermore, we identified negative correlations between epigenetic regulators and the lipid metabolism drivers *SMUG1* and *CDS1*, with cbFFA. A set of placental miRNAs showed positive correlations with cbFFA. Using mirTarRnaSeq, an R/Bioconductor package, we predicted interactions between placental coding genes and miRNA, which correlated negatively and positively with cbFFA, respectively. Several FFA-associated miRNAs (miR-23b cluster, -168, -138, -6825, -6845) have been previously associated with obesity in animal models and human cohorts.

**Discussion:**

Further studies are required to investigate the role that the Rho network plays in placental inflammation and the link between miRNAs and the predisposition towards cardiovascular diseases in the offspring of obese mothers.

## Introduction

Obesity in pregnancy has reached epidemic levels in the US where almost one third of reproductive age women are affected by obesity prior to becoming pregnant (1). Maternal obesity (pregravid body mass index (BMI) ≥30kg/m^2^) not only affects maternal outcomes but exposes fetuses to an intrauterine milieu that alters development and impacts offspring long-term disease risk. Indeed, offspring of mothers affected by obesity have a greater risk of obesity (2), non-alcoholic fatty liver disease (NAFLD) (3) and overall, are affected by higher rates of cardiovascular disease (CVD) (4, 5) and all cause premature deaths (5). The placenta plays a key role in fetal development, controlling access to nutrients, essential for normal tissue growth. Evidence has shown that maternal obesity alters lipid transport mechanisms (6) into and out the placenta and promotes placental lipotoxicity (7). In addition, obesity triggers low-grade inflammation in the placenta with an elevated presence of immune cells, mostly macrophages (8). This inflammatory phenotype may be connected to changes in lipid metabolism (8), however, the pathways that mediate the placental response to altered lipid metabolism in maternal obesity and the molecular link with inflammation remain largely unknown.

MicroRNAs (miRNAs) are small non-coding RNAs that bind to mRNA, repressing translation to protein and leading to transcript degradation; they are important regulators of placental development and function (9). Previous studies (10, 11) reported alterations in placental miRNAs in mothers with obesity, some which may be connected to fetal growth outcomes (12). However, in these studies, patients enrolled as obese often present with pregnancy associated co-morbidities, such as preeclampsia and gestational diabetes (10, 11) making it difficult to tease out the impacts of maternal BMI vs concurrent morbidities. In addition, the focus on studying the effects of low vs high BMI does not capture the complex pathway interactions that occur in the placenta and may drive pregnancy outcomes. This is important, as infant outcomes vary within obese pregnancies, and may depend on placental response to the maternal metabolic milieu.

To address the above gaps, we conducted a global, unbiased correlation analysis between placental transcripts and metabolic traits of healthy mothers affected by obesity and their offspring. Our novel approach aims to understand the placental molecular dynamics in obese patients, and specifically to identify mRNA-miRNA interactions associated with the metabolic milieu in the mother-infant dyad.

## Methods

### Data collection

Data and samples used in this study were collected as part of a prospective cohort of women delivering via scheduled cesarean from 2004- 2016. Maternal demographics, height and weight were obtained following written informed consent as approved by the Institutional Review Board at MetroHealth Medical Center in Cleveland Ohio (IRB #1300650). Net maternal gestational weight gain was calculated as total pregnancy weight gain minus birth weight and placental weight. Placental tissue was collected within 30 minutes of delivery, weighed, and sampled immediately. Samples were collected below the placental basal plate, avoiding any macroscopic lesions, and further dissected into small pieces, blotted for blood removal and snap frozen in liquid nitrogen. Within 48 hrs. of birth, neonatal anthropometrics were measured and recorded by a trained research nurse. Birth weight was measured on a calibrated scale, and a measuring board was used for length measurements. The flank skinfold was assessed in the mid-axillary line directly above the iliac crest. Neonatal body composition estimates were made using the following validated equation: fat mass (FM) = 0.39055 (birth weight, kg) + 0.0453 (flank skinfold, mm) – 0.03237 (length, cm) + 0.54657. Lean body mass (LBM) was calculated as birth weight minus fat mass. Percent body fat (neoadp) = fat mass/birth weight x 100.

### Sample selection and grouping

Placental samples were chosen for transcriptomic profiling based on maternal pre-pregnancy body mass index (BMI) and neonatal adiposity (% body fat). Samples were included if pre-pregnancy BMI ≥ 30 kg/m^2^. Further, samples were divided into tertiles by neonatal adiposity based upon % body fat at birth. The first (low adiposity, LA) and third (high adiposity, HA) tertiles were chosen for further analysis, creating two groups: obese women with low (OBLA, N = 20) and high (OBHA, N = 19) adiposity offspring.

### Placental RNA isolation

Total placental RNA was extracted by homogenization of ~50 mg placental tissue in TRIzol reagent (Invitrogen, Carlsbad, CA), following the manufacturer’s guidelines. RNA quantification and integrity were measured using an Agilent 2100 Bioanalyzer (Agilent, Santa Clara, CA). All samples had an RNA integrity number > 7.

### Transcriptomic profiling

Library preparation and sequencing was conducted at Azenta Life Sciences (South Plainfield, NJ, USA) as follows: Library Preparation with PolyA selection and Illumina Sequencing RNA samples were quantified using Qubit 2.0 Fluorometer (Life Technologies, Carlsbad, CA, USA) and RNA integrity was checked using Agilent TapeStation 4200 (Agilent Technologies, Palo Alto, CA, USA). RNA sequencing libraries were prepared using the NEBNext Ultra II RNA Library Prep for Illumina using manufacturer’s instructions (NEB, Ipswich, MA, USA). Briefly, mRNAs were initially enriched with Oligo d(T) beads. Enriched mRNAs were fragmented for 15 minutes at 94°C. The first strand and second strand cDNA were subsequently synthesized. cDNA fragments were end repaired and adenylated at 3’ ends, and universal adapters were ligated to cDNA fragments, followed by index addition and library enrichment by PCR with limited cycles. The sequencing libraries were validated on the Agilent TapeStation (Agilent Technologies, Palo Alto, CA, USA), and quantified by using Qubit 2.0 Fluorometer (Invitrogen, Carlsbad, CA) as well as by quantitative PCR (KAPA Biosystems, Wilmington, MA, USA). The sequencing libraries were clustered on the HiSeq (4000 or equivalent) flowcell. After clustering, the flowcell was loaded on the Illumina instrument according to manufacturer’s instructions. The samples were sequenced using a 2x150bp Paired End (PE) configuration. Image analysis and base calling were conducted by the Control software. Raw sequence data (.bcl files) generated by the sequencer were converted into fastq files and demultiplexed using Illumina’s bcl2fastq 2.17 software. One mismatch was allowed for index sequence identification.

### WGCNA

WGCNA for coding genes and miRNAs was run separately for the different level of gene expression, with coding genes on average having higher level expression than miRNAs. This pushed us to adopt different filter masks to remove lower values after normalization (see details below). In addition, since WGCNA clusters genes into modules according to expression patterns, we were concerned that potential functional relationships between coding and non-coding elements may have altered gene distribution therefore modules formation. Signed networks were created for the WGCNA analysis.

### Protein coding genes

For normalization, gene expression data were converted into a matrix, including maternal age, race, smoking, trimmed placenta weight, fetal sex, group, and gene ID, which were subsequently excluded from the final heatmap. The row names of the matrix were set to the gene ID values extracted from the gene expression dataset. We created DESeq2 dataset `dds` with the expression matrix and sample table and ran the normalization using regularized Log Transformation (rlog). We filtered out gene counts lower than 1 prior to analysis. To further minimize noise, we created a filter mask (greater and equal to 1^st^ Q) to remove the lower values after normalization. Briefly, 1. we chose soft-thresholding powers within the range of 1 to 20 and set the model complexity to verbose=3 for topology analysis. 2.Plotted the scale Independence and Mean connectivity to find the elbow point. We kept the scale of independence at 0.85. The number that reached or surpassed the line indicated the power value suitable for the network. 3. The selected power was 1.0. We combined similar modules with a threshold of 0.25 and visualized in dendrograms. To calculate the correlation between module eigengenes and traits, the expression data matrix and the merged color module were first used to calculate the eigenvalues. The row names of the resulting eigenvalues were matched with those of the expression data to prepare for matching the trait data. The intersection of sample names between eigenvalues and traits was then found to ensure that only common samples were used in the correlation analysis. The matched eigenvalues and traits were used to calculate the correlation between module eigengenes and trait data. P-values for the correlation results were calculated using the corPvalueStudent function to determine statistical significance. Finally, the correlations and p-values were visualized using the pheatmap function. The same method was used to generate a heatmap to visualize the correlation between modules.

miRNAs: Normalization for miRNAs was performed similarly to coding genes, except that variance stabilizing transformation (VST) was used instead of rlog normalization. This adjustment was made to reduce variance, as the miRNA dataset was smaller and right skewed.

We created a filter mask (greater and equal to 2^nd^ Q) to remove lower values after normalization to further avoid noise in the dataset. Briefly, the preparation was similar to those used for coding genes, except the selected power for miRNA analysis was 4. Furthermore, modules were merged with a threshold of 0.65, compared to 0.25 for coding genes.

### Reactome

We used the open-source, manually curated and peer-reviewed Reactome database to identify molecular pathways within selected modules: Red, Purple, Green, Brown, Tan. All the Pathways with a p-value <0.05 were considered significant and reported. In addition, the genes included in modules Brown, Red and Purple were also combined and analyzed together to explore positive correlations between maternal (maternal Leptin) and fetal (cord blood FFA) lipid profiles with placental gene expressions.

### mirTarRNASeq

Selected coding genes from the MEgreen and MEtan modules and miRNAs from the MEgreen and MEturquoise module were used for this analysis. The *mirTarRNASeq* package in R was utilized to process and analyze the data. Gene expression values were normalized and rounded to integers to meet the statistical requirements of the *mirTarRNASeq* model. Using the *mirTarRNASeq* function *combiner*, the protein-coding and miRNA datasets were merged to facilitate the comparison of miRNA and protein-coding expression profiles across the same samples. Next, the *geneVari* function was then applied to calculate gene variance, ensuring the inclusion of variable features for subsequent modeling. Genes from the protein-coding dataset were selected based on molecular function in the biological context of this study. The Poisson regression model was employed to analyze the relationship between miRNA and protein-coding gene expression. The *mirTarRNASeq* function *runModels* was used to fit the models, specifying a Poisson family distribution and a scaling factor of 100 to standardize the model coefficients. Each protein-coding gene was modeled iteratively, and the results were stored in a list of model summaries. The *blaPois* method was chosen for its efficiency in handling multiple models, including *blaGaus, blaNB, blazeroinflNB*, and *blazeroinfl*, offering the ability to process expression datasets with relatively low dispersion. This approach was particularly effective for datasets with low expression counts, where each expression occurred independently. To minimize potential errors during model fitting, the *try* function was implemented, ensuring uninterrupted execution. The model summary included the gene ID, the estimate, standard error, z-value, and p-value associated with each gene ID.

## Results

### Several metabolic and clinical traits of the maternal-fetal dyad correlated with placental gene expression in the setting of obesity

**Figure 1:**
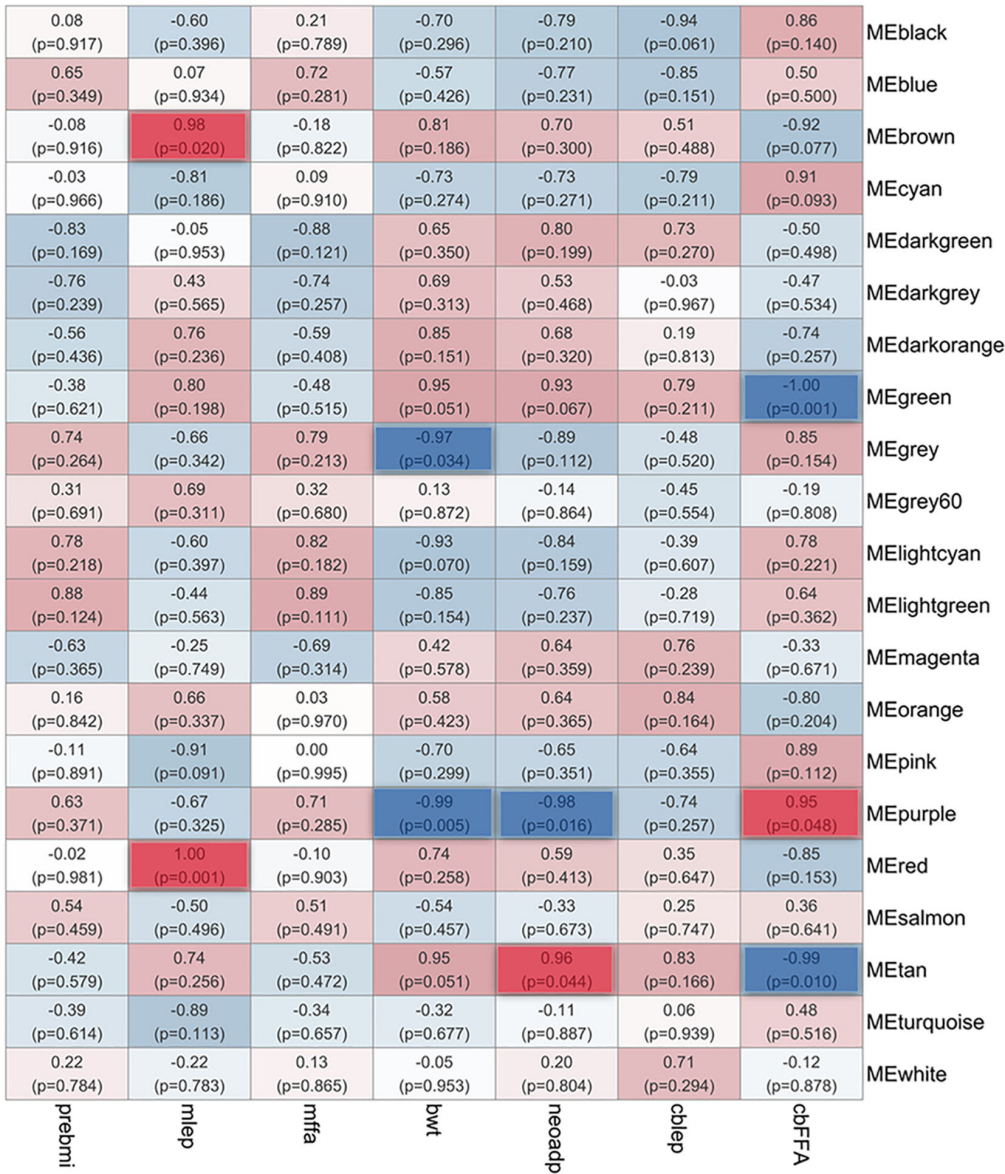
Coding gene modules-metabolic traits correlations in placentas from pregnancies affected by obesity. Using Whole Genome Correlations Network Analysis (WGCNA), we established correlations between placental coding-genes with similar patterns of expression (Modules) with metabolic traits of the Maternal-Fetal dyad. Each correlation contains a coefficient of correlation and a (p-value). Red correlations are positive, Blue correlations are negative. Highlighted RED: significant positive correlations; Highlighted Blue: significant (p <0.05) negative correlations. cbFFA, cord blood Fatty-Acids; cblep cord blood leptin; neoadp, neonatal adiposity; bwt, birth weight; mffa, Maternal Free Fatty-Acids; mlep, Maternal Leptin; preBMI, Maternal Pre-pregnancy BMI.

Using Next generation Sequencing (NGS) data from N = 39 placentas collected at cesarean delivery from women with a pregravid BMI ≥ 30 kg/m^2^ (**Table 1**) we performed a Whole Genome Correlation Network Analysis (WGCNA) (13) to establish, in a global and unbiased modality, correlations between clinical and metabolic traits of the maternal-fetal dyad with patterns of placental gene expression. In the WGCNA analysis, genes with similar patterns of expression form modules (ME), the correlation of the ME with specific variables produces a module-trait correlation matrix (**Figure 1**). Each correlation presents a coefficient of correlation (ρ) and a p-value. We considered correlations significant if ρ was higher or lower than +/- 0.5 and a p-value ≤ 0.05. Overall, we identified 17 significant correlations between modules and the clinical and metabolic traits analyzed (12 negative and 5 positives; not all data are shown in **Figure 1**). Of the data not shown in **Figure 1**, the following correlations were found: MEpurple showed negative correlation with smoking (ρ=-0.97; p-value=0.025) and negative correlation with gestational age (GA) (ρ=-0.97, p-value=0.025). Interestingly, MEtan and MEpurple showed significant but inverse correlations with cord blood Free-fatty Acids (cbFFA) (ρ=-0.99, p-value=0.01; ρ=0.95; p-value=0.048) and neonatal adiposity (neoadp) (ρ=0.096, p-value=0.04; ρ=-0.98, p-value=0.016). MEpurple showed also a significant negative correlation with neonatal birth weight (bwt) (ρ=-0.99; p-value=0.005). MEdarkorange showed a negative correlation with maternal *homeostatic model assessment* (mHOMA) (ρ=-0.99; p-value=0.01). MEgrey showed negative correlations with placental weight (ρ=-0.99,p-value=0.015) and bwt (ρ=-0.97; p-value=0.034). MEdarkgreen showed a positive correlation with maternal age (ρ=0.97 p-value=0.031). MElightcyan and MElightgreen were negatively correlated with placental weight (ρ=-0.98; p-value=0.015; ρ=-0.97, 0.035 respectively). MEwhite showed negative correlation with birth length (blg) (ρ=-0.96; p-value=0.035). Neither race nor pre-pregnancy BMI showed any significant correlations with placental gene expression among obese patients. All genes contained in modules with significant correlations are listed in **Supplementary Material 1** WGCNA.

**Table 1:** Maternal and neonatal variables.

Maternal variables	n	Mean±SD
Age, yr	39	27.8±5.8
Pre-pregnancy BMI (kg/m^2)	39	34.2±3.1
Race (NHB/NHW)	39	19/20
Smoking (N/Y)	39	32/7
Leptin, ng/mL	34	65.9±24.5
FFA, mEq/L	36	0.83±0.26
Insulin, uU/mL	35	18.5±6.9
Glucose, mg/mL	39	77.6±7.9
HOMA-IR	35	3.5±1.5
Neonatal variables	n	Mean±SD
Gestational age, wk	39	38.9±0.3
Sex (F/M)	39	21/18
Birth weight, kg	39	3.3±0.5
Birth length, cm	39	49±1.7
Neonatal Adiposity %	39	12.3±4.3
Untrimmed placental weight, gr	39	673±202
Leptin ng/mL	27	15.3±13.6
FFA, mEQ/L	37	0.5±1.9
Insulin, uU/mL	37	8.18±4.3
Glucose, mg/mL	37	64.8±12
HOMA-IR	35	1.2+0.6

NHB, non-Hispanic Blacks; NHW, non-Hispanic White; FFA, Free Fatty-Acids; HOMA-IR, Homeostatic Model assessment of Insulin Resistance.

### Placental expression of members of the Rho network correlate positively with maternal leptin and cord blood free fatty acids

Evidence has shown that maternal obesity alters intrauterine lipid metabolism and promotes placental lipotoxicity (7). Furthermore, lipid profiles in the cord blood are associated with neonatal adiposity (14), the latter an early marker of metabolic syndrome in the offspring of mothers affected by obesity. For these reasons, given the strong correlation shown in our analysis between placental gene expression with maternal leptin and cbFFA (**Figure 1**), we investigated the molecular pathways associated with genes contained in the modules MEbrown and MEred correlating with maternal leptin, and MEpurple with cbFFA. Using Reactome (15), an open-source, peer-reviewed pathway database, we characterized genes in modules-of-interest according to their functional pathways. Only pathways that presented a significant p-value (<0.05) are listed (**Tables 2**, **3**; **Supplementary Table 1**). Significantly overrepresented pathways in the MEred module, which contains 1,107 genes and was positively correlated with maternal leptin, are shown in **Table 2**. Genes belonging to the “Nuclear Pore Complex” and “Regulation of Glucokinase Regulatory Pathways” participate in intracellular trafficking in basic cellular activities like cell cycle and metabolism. Interestingly, *MECP2* and its co-repressor *SIN3A* are found in this module. These genes play a fundamental role in securing genomic areas that have been previously methylated, participating in gene silencing (16). Also positively correlated with maternal leptin, the MEbrown module (**Supplementary Table 1**) contains 1,782 genes. The most highly represented pathway in this module involves “Antigen Processing: Ubiquitination and Proteasome Degradation” with more than 50 genes including many Ubiquitin-like proteins (UBE) and Ubiquitin-associated proteins (UBA). Eight genes represented pathways involved in spatial chromatin organization (“Cohesin Loading onto Chromatin;” “Establishment of Sister Chromatid Cohesion”*)*. Several members of the Nucleoporin (NUP) family are represented in the “Regulation of Glucokinase by Glucokinase regulatory protein” pathway. Finally, the MEbrown module contained *CDC42, CDC42SE1* and *DOCK11*, all members of the Rho family. The MEpurple module (**Table 3**) correlated positively with cbFFA and contained only 574 genes. This module’s most represented pathway was “Neutrophil degranulation” which included genes known to be involved in neutrophil activity. The MEpurple module also contained several pathways directly connected with the Rho family including *CDC42*. Other notable pathways represented in this module are associated with Caspase activity (“*CASP8* activity is inhibited”), Extracellular Matrix organization (“Elastic Fiber Formation”) and metabolism of proteins (“Post-Translational protein Phosphorylation”).

**Table 2:** Reactome analysis of the coding-gene module “MEred’ in placentas obtained from mothers affected by obesity.

System	Pathway	Significant genes	P-value	FDR
Signal Transduction	RHOQ GTPase cycle; CDC42 GTPase cycle; RHOj GTPase cycle; RHOG GTPase cycle; RHOD GTPase cycle; RAC1 GTPase cycle; Competing Endogenous RNAs (ceRNAs); Negative Regulation of TCF-dependent signaling by DVL-interacting proteins.	CDC42BPB, RAB7A, CDC42EP4, RHOB ARHGAP17-30-21, ARHGEF11-12-5, PAK4, DOCK1-8,	0.00049	0.86
Gene Expression	Post-Transcriptional silencing by small RNAs.; Regulation of NPAS4 mRNA Translation; RNA Polymerase II Transcription Termination; Small Interfering RNA (siRNA) Biogenesis; Regulation of Endogenous retro elements by Piwi-interacting RNAs (piRNAs)	AGO1, TNRC6A	0.0014	0.99
Cell Cycle	Nuclear Pore Complex (NPC) Disassembly;	NUP98-188-214, POM121C-121	0.0027	0.99
Disease	Defective TPR may confer susceptibility towards Thyroid papillary carcinoma (TPC); Loss of MECP2 binding ability to 5mC-DNA; Disorders of Developmental Biology; Disorders of SNC;	MECP2, SIN3A, NCOR1	0.003	0.99
Metabolism	Regulation of Glucokinase Regulatory Protein	NUP98-188-214, POM121C-121	0.008	0.99
Cell-cell Communication	Regulation of CDH11 mRNA translation by microRNAs	AGO1, TNRC6A	0.01	0.99

Using Reactome, an open source, peer-reviewed pathway database, we analyzed genes contained in the MEred module positively correlating with maternal leptin in the WGCNA analysis. The p-value and FDR reported belong to the pathway with the most significant p-value listed in that specific system. The pathways reported are all the pathways that presented p-value <0.05 in the Reactome analysis for all the genes listed in Mered.

**Table 3:** Reactome analysis of the coding-gene module “MEpurple” in placentas obtained from mothers affected by obesity.

System	Pathway	Significant genes	P-value	FDR
Immune System	Neutrophil Degranulation; Endosomal/Vacuolar Pathway; IL-21 signaling;	HLAB-C, MMP9, IGFR2, MAPK1, CTSA, SLC2A3, TMC6, TSPAN9-14	0.0004	0.13
Signal Transduction	CDC42 GTPase cycle; RHOQ; RAC1; RHOC; RHOB; RHOJ; RAC3; RAC2; RHOA	CDC42EP1-3-5, ARHGAP1-33, ARHGDIA, ARHGEF10-25, GIT1-2, ROCK1, GIT1-2, FNBP1	0.0002	0.13
Neuronal System	Tandem of pore domain in a weak inwardly rectifying K+ channels;	KCNK6-7	0.008	0.98
Programmed cell Death	CASP8 activity is inhibited	FAS, CFLAR	0.01	0.98
Developmental Biology	SEma4D induced cell migration and growth-cone collapse; Sema4D in semaphoring signaling;	MYH9, MYL9, SEMA4D	0.01	0.98
Extracellular Matrix Organization	Elastic Fiber Formation; Molecules Associated with elastic fibers;	TGFB3, LOXL2, BMP2, EFEMP2	0.012	0.98
Metabolism of proteins	Post-translational protein phosphorylation	IGFBP3-7, SPARC, TIMP1, APOE	0.015	0.98
Disease	Defective NEU1 causes Sialidosis	GLB1L, CTSA	0.03.2	0.98

Using Reactome, an open source, peer-reviewed pathway database, we analyzed genes contained in the MEpurple module positively correlating with cbFFA in the WGCNA analysis. The p-value and FDR reported belong to the pathway with the most significant p-value listed in that specific system. The pathways reported are all the pathways with a p-value <0.05 in the Reactome analysis for all the genes listed in MEpurple.

**Figure 2:**
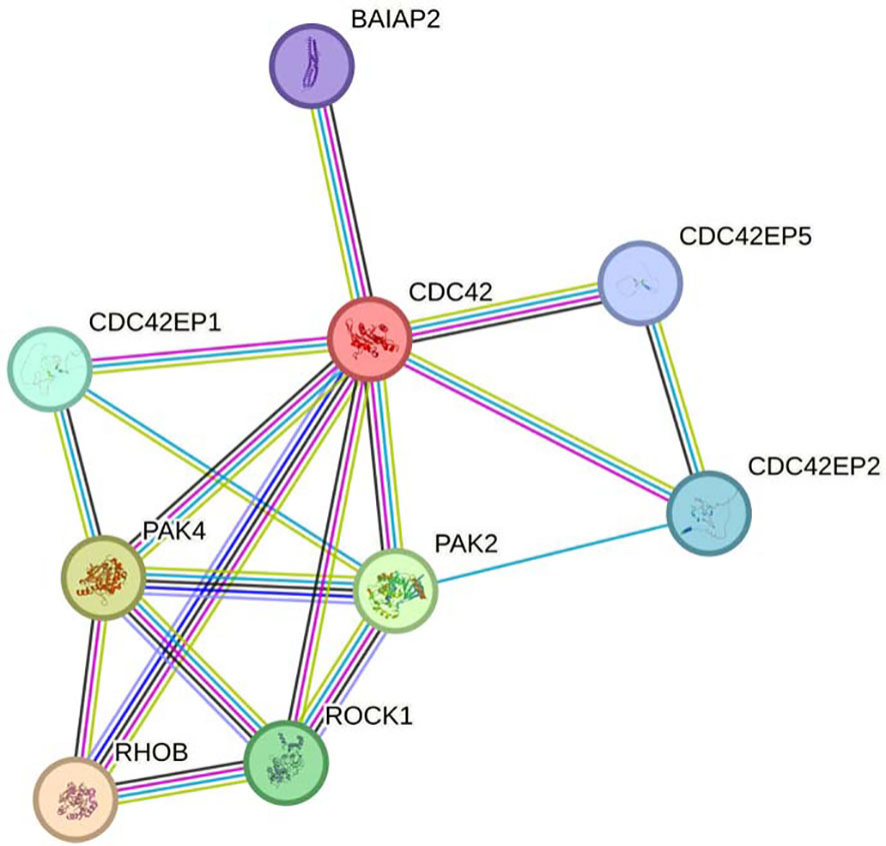
String analysis of the RHO GTPases network components (GTPases + effectors) identified in MEred, MEbrown and MEpurple modules. Note the central functional position occupied by CDC42. Colored nodes: query proteins and first shell of interactors. 
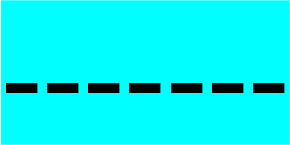
 line: Curated database. 
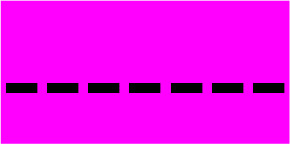
 line: Experimentally determined. 
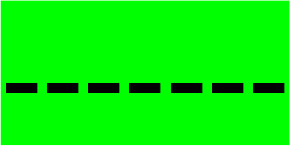

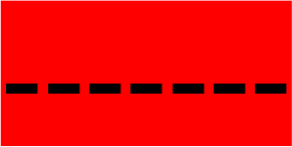

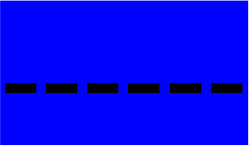
 lines: Predicted interactions.

Collectively, these data show a clear overrepresentation of genes that are part of the Rho GTPases family or have functional association with it. Interestingly, when the genes of the 3 modules (MEred, MEbrown and MEpurple) that correlated positively with either maternal leptin or cbFFA are analyzed together, using Reactome, the only significant pathways identified are Rho GTPases-associated. Rho GTPases, represented in these modules by *RhoB, RIF1* (MEbrown) and *CDC42* (MEred), operate within a complex system of activators (called guanine nucleotide exchange factor, GEFs), inhibitors (called GTPase-activating proteins, GAPs), and surrogate proteins that mediate Rho GTPases activity, several of these are represented in this analysis (**Supplementary Table 2**) (17). The functional interconnectivity of all these elements emerges with a string analysis (**Figure 2**) that showed the network structure and the central role of CDC42.

### Placental expression of components of the epigenome and lipid metabolism correlate negatively with free fatty acids in the cord blood

**Figure 3:**
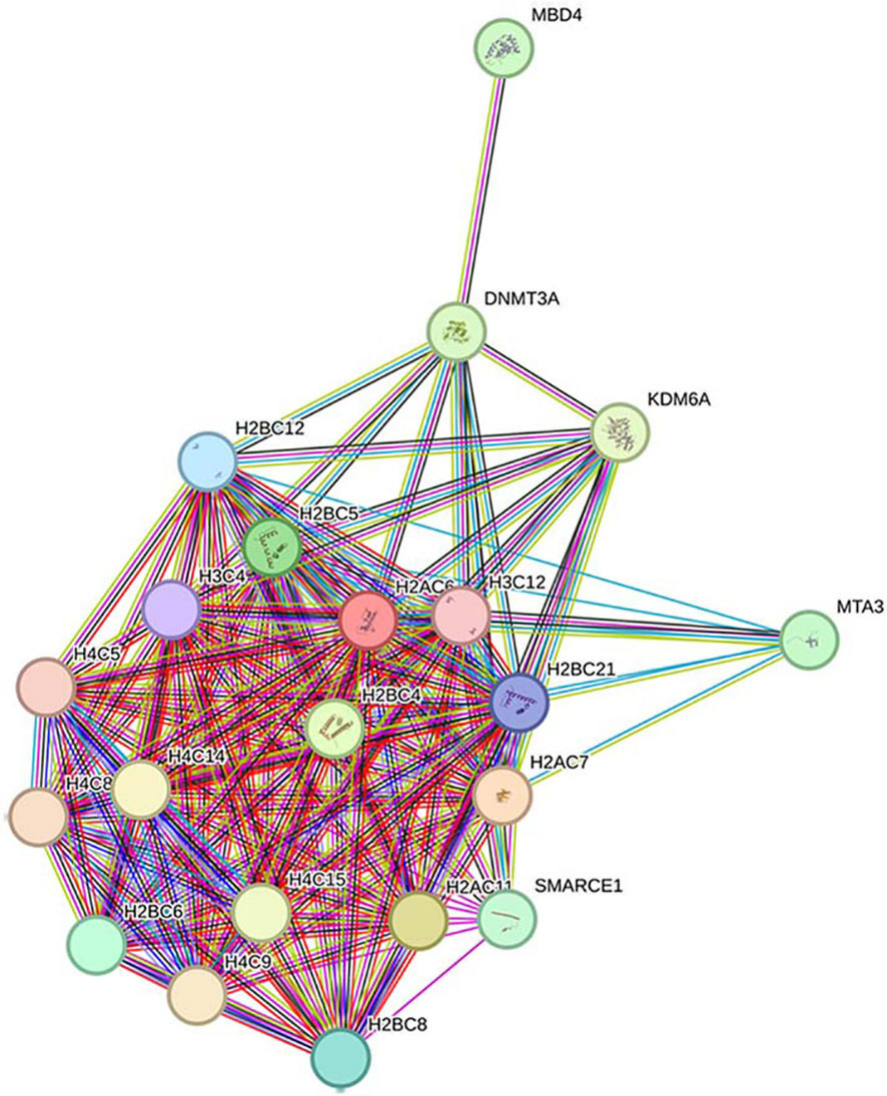
String Analysis of selected components of the Epigenome expressed in the MEgreen. Colored nodes: query proteins and first shell of interactors. 
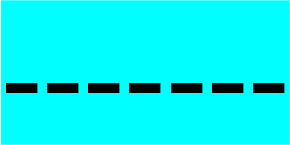
 line: curated database. 
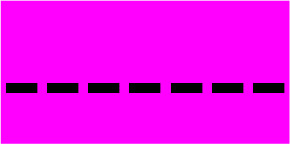
 line: Experimentally determined. 
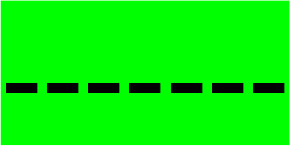

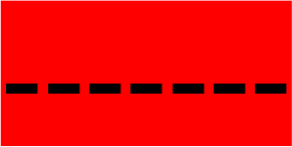

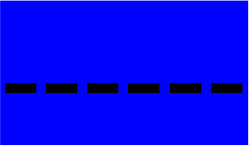
 lines: predicted interactions.

Applying the same method as above, we identified overrepresented pathways within MEs that *negatively* correlated with cbFFA - MEgreen and MEtan (**Figure 1**). In the green module (**Table 4**), we observed that most of the overrepresented pathways operate in the cell nucleus and were associated with gene transcription systems, such as “PRC2 Methylates histones and DNA”, “RNA Polymerase I promoter opening”, “Transcription regulation by small RNAs”. In the DNA Repair and, Disease and Chromatin organization systems we found “Cleavage of the Damaged Pyrimidine,” “Recognition and Association with DNA Glycosylase containing affected Purine,” “Cleavage of Damage Purine;” “Defective Pyrotosis;” “HDACs” (**Table 4**). Globally, the total number of genes that populate these pathways is modest (36 genes in total) (**Supplementary Table 3**). Around 60% (22/36) of these genes are components of the epigenome (**Supplementary Table 3**), including various isoforms of the core histones (*H1-2, H2AC6-7-11, H2BC4-5-6-8-12-21,H3C4-12, H4C5-8-9-14-15*), *DNMT3A, MBD4* and chromatin remodelers such as *MTA3, SMARCE1* (**Figure 3**). Interestingly, they represent key elements of the epigenetic machinery. This module also contains four individual genes involved in lipid metabolism. *SMUG1*, an enzyme specialized in DNA repair, has been associated with non-alcoholic fatty liver disease in morbidly obese individuals (18) while *CDS1* is a member of CDP diacylglycerol synthase.

**Table 4:** Reactome analysis of the coding-gene module “MEgreen’ in placentas obtained from mothers affected by obesity.

System	Pathway	Significant genes	P-value	FDR
Gene Expression	PRC2 Methylates histones and DNA; RNA Polymerase I Promoter Opening; Regulation of the Endogenous Retroelements by the HUSH complex; RNA Polymerase I Promoter Escape; DNA Methylation; Regulation of Endogenous Retro elements by piRNAs; Transcription regulation by small RNAs	DNMT3A, MBD4, MTA3, RSF1, SMUG1, CDS1, KDM6, SMARCE1 H1-2, H2AC6-7-11, H2BC4- 5-6-8-12-21,H3C4-12, H4C5-8-9-14-15	0.0000002.7	0.00026
DNA Repair	Cleavage of the damaged Pyrimidine; Recognition and Association with DNA Glycosylase containing affected Purine; Cleavage of damage Purine; Depurination; Recognition and Association of DNA Glycosylase with site containing an affected Pyrimidine; Depyrimidination; Base Excision Repair;	H2AC6-7-11, H2BC4-5-6- 12, H1-2	0.0000003.6	0.00026
Disease	Defective Pyrotosis	H2BC4-5-6-12, H1-2	0.000002.2E	0.0012
Chromatin Organization	HDACs (histone deacetylate)	H2AC6-7-11, H2BC4-5-6- 12, H1-2 H4C5-8-9	0.00001	0.0025
Cell Cycle	Nucleosome Assembly; deposition of new CENPA containing nucleosome at the centromere; Packaging of Telomere Ends; G2/M DNA Damage Checkpoint	H2AC7, H2BC4-5-6, H4C8- 9-14-15	0.00001.6E	0.0031
Developmental Biology	Chromatin Modifications during the Maternal to Zygotic Transition (MZT)	H2AC7, H2BC5-6-8-12- 216KDM6A	0.0001.33	0.0182
DNA Replication	Assembly of the ORC Complex at the origin of Replication;	H2BC12-21, KPNB1	0.0001.5	0.019

Using Reactome, an open source, peer-reviewed pathway database, we analyzed genes contained in the MEgreen module negatively correlated with cbFFA in the WGCNA analysis. The p-value and FDR reported belong to the pathway with the most significant p-value listed in that specific system. The pathways reported are all the pathways with a p-value <0.05 in the Reactome analysis for all the genes listed in MEgreen.

### cbFFA correlates with placental miRNA expression in mothers affected by obesity

To identify placental miRNAs associated with maternal and neonatal metabolic traits of interest and coding gene modules, we used the same global and unbiased approach used for coding genes. Using WGCNA, we established correlations between miRNA expression and fetal-maternal traits (**Supplementary Figure 1**). Interestingly, cbFFA showed significant positive correlations with MEturquoise (ρ=0.97; p-value=0.029) and MEgreen (ρ=1; p-value=0.000) miRNA modules, which together contain 241 miRNAs. MEblue showed significant positive correlation with mLep (ρ=0.97; p-value=0.042). Two significant negative correlations were identified between MEturquoise with birth weight (ρ=-0.98; p-value=0.025) and MEred with cbFFA (ρ=-0.96; p-value=0.037).

### miRNA and coding genes show potential interactions in placentas of mothers affected by obesity

**Figure 4:**
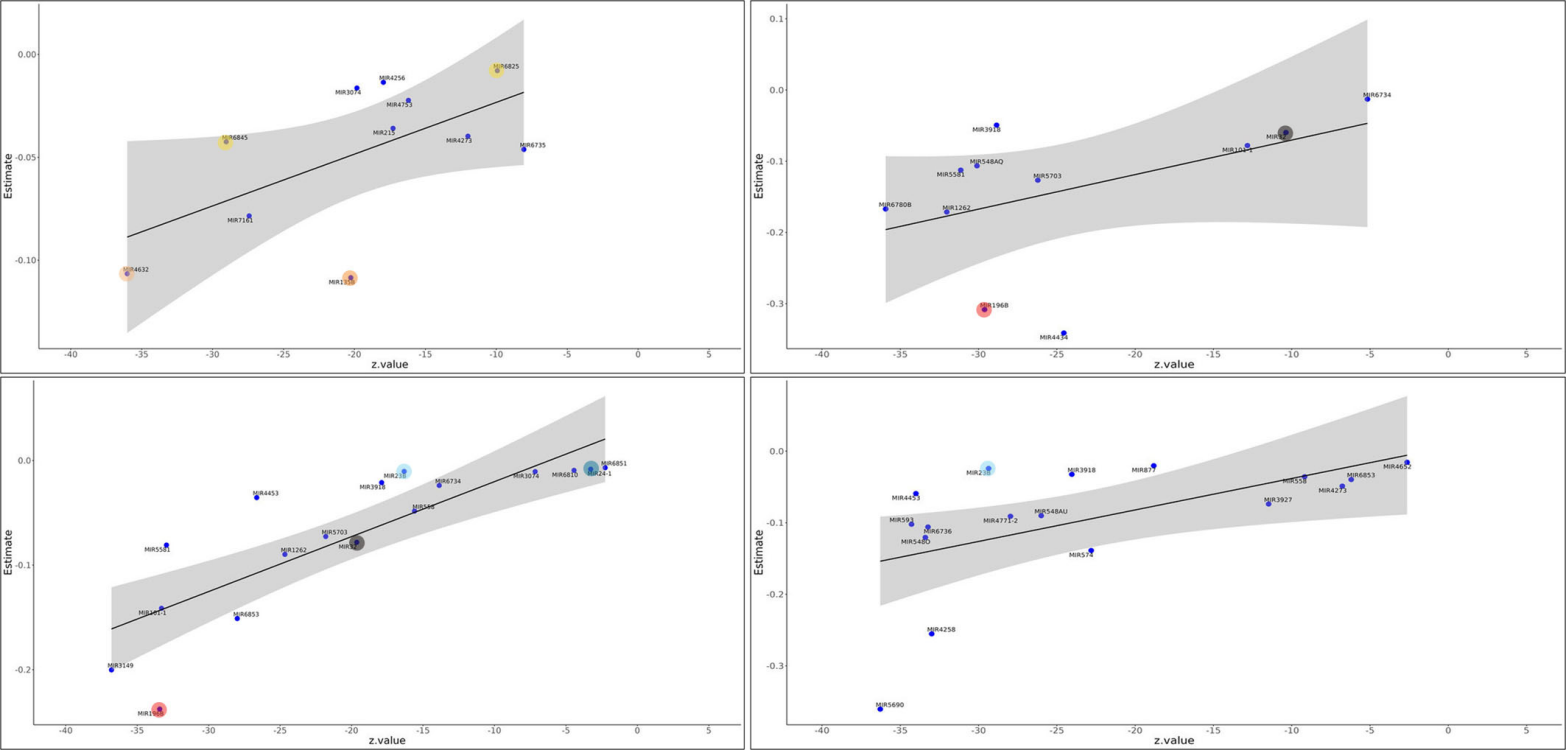
Panels **(a–d)** Linear regression between components of the methylation machinery and miRNAs. Using mirTarRnaSeq we predicted linear regression between miRNAs in the MEgreen+METurquoise modules (positively correlated with cbFFA) and components of the Epigenetic machinery contained in the MEgreen module (negatively correlated with cbFFA). All the miRNA-gene interactions reported have a p-value <0.05. Panel **(A)**, upper left DNMT3A; Panel **(B)**, upper right MBD4; Panel **(C)**, lower left MTA3; Panel **(D)**, lower right KDM6. Yellow: miR-6845 and -6825 previously identified in the screening for obesity in pediatric populations; Orange: miR-4632 and Dark Orange: miR-135b have been identified in experimental models of obesity [37]; Red: miR-196b, this miRNA is considered a master regulator of adipogenesis Azure: miR-23b. This gene is known to regulate KDM6 and it is overexpressed in obesity [40]; Dark Azure: miR-24, was overexpressed in livers of mice with high fat diet; Red: miR-196b, this miRNA is considered a master regulator of adipogenesis; Black: miR-32 was shown to be overexpressed in patients with hepatic steatosis.

**Figure 5:**
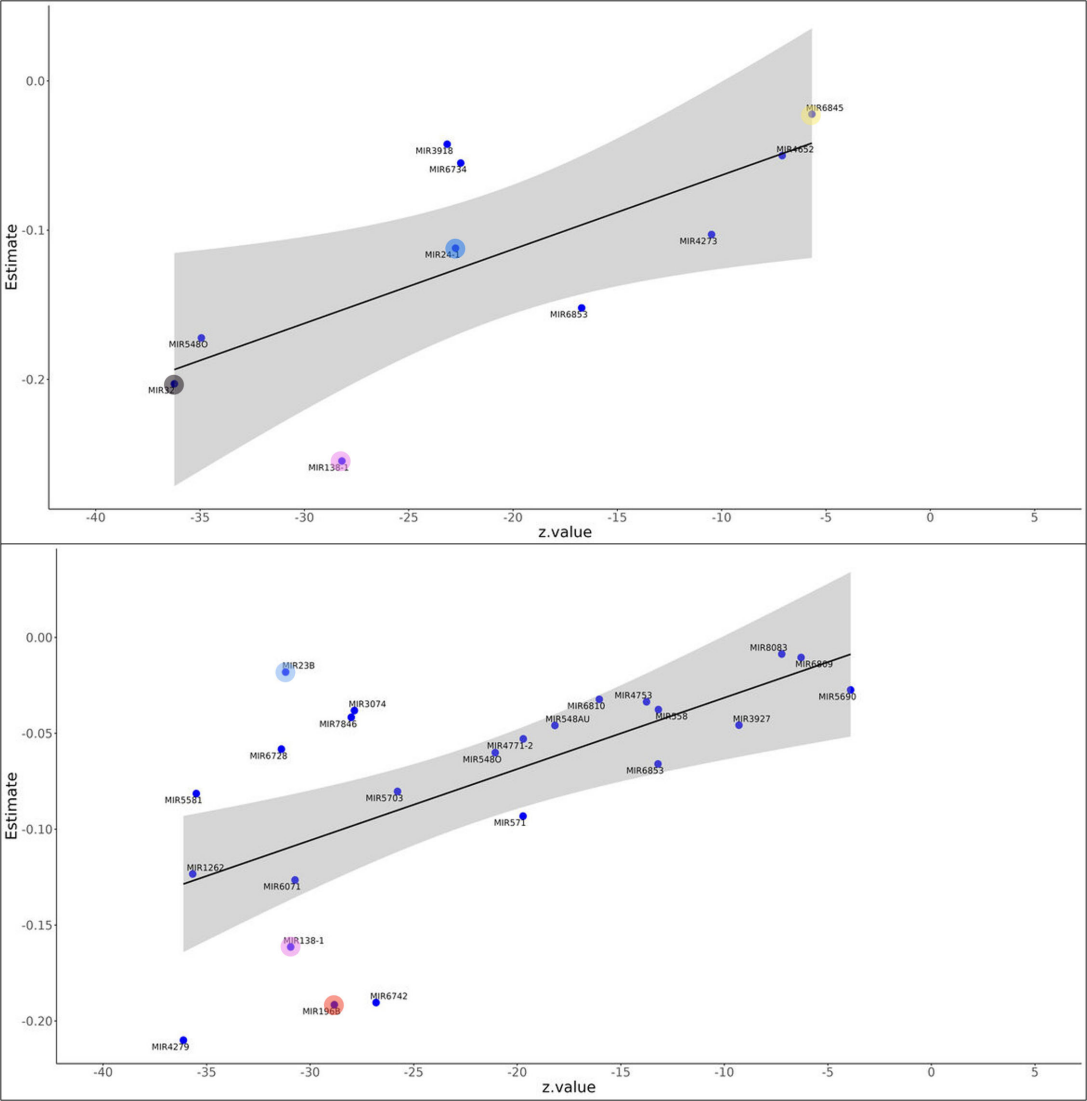
Panels **(a, b)** Linear regression between Lipid metabolism genes and miRNAs. Using mirTarRnaSeq we predicted linear regression between miRNAs in the MEgreen+METurquoise modules (positively correlated with cbFFA) with genes involved in Lipid metabolism contained in the MEgreen module (negatively correlated with cbFFA). All the miRNA-gene interactions reported have a p-value <0.05. Panel **(A)**, upper CDS1; Panel **(B)**, lower SMUG1. Azure: miR23b. This gene is known to be overexpressed in obesity; Red: miR-196b, this miRNA is considered a master regulator of adipogenesis; Pink: miRNA-138 this miRNA is closing involved in targeting adipogenesis Lipoprotein Lipase, a key lipogenic enzyme in ahMSCs. The datasets for this study can be found in the Harvard Dataverse: https://doi.org/10.7910/DVN/LOIDQS.

We hypothesized that coding gene modules (MEgreen and MEtan) negatively correlated with cbFFA (**Figure 1**) may have functional relationships with miRNA modules (METturquoise and MEgreen), which were positively correlated with the same metabolic trait (**Supplementary Figure 1**). To predict these interactions, we used mirTarRnaSeq (19) an R/Bioconductor package for quantitative assessment of miRNA-mRNA interactions. We combined and analyzed the two miRNA modules (MEturquoise and MEgreen) with selected coding genes in modules (MEgreen and MEtan) separately. For the MEgreen cohort, we selected those 37 genes contained in the Reactome pathways with significant p-value (the genes are listed in **Supplementary Material 1**). Overall, we performed 2,923 regressions analyses, excluding those with a P-value >0.05, resulting in 1,252 regressions (all listed in **Supplementary Material 1**). Further filtering for z-score less than -2 reduced the dataset to 633 significant miRNA-mRNA regressions for all the coding genes in MEgreen. Among these, we highlighted specific mRNA-miRNA interactions (see **Figures 4**, **5**). In the MEgreen coding genes module, we focused on 3 different classes of epigenetic regulators: regulators of DNA methylation (*DNMT3A and MBD4);* chromatin remodeler (MTA3) and histone modifier (KDM6). *DNMT3A* and *MBD* cooperate to silence functional areas of the genome. *DNMT3A* and *MBD4* had 11 significant linear regressions (**Supplementary Material 1**) but none with the same miRNA (**Figures 4A, B**). None of the miRNAs identified in this analysis were previously reported targeting *DNMT3A* and *MBD4*. miR-135b has been described in experimental models of obesity (z-score-20, P-value 3.03E-91). *MTA3* and *KDM6* showed significant regressions with 18 miRNAs each (**Figures 4C, D**). Interestingly, miR-23b showed a significant interaction with both genes (*MTA3* z-score -29, p-value=1.8E-189; *KDM6* z-score -16, p-value=6.91E-60).

*SMUG1* and *CDS1*, genes involved in lipid metabolism, present 11 and 25 significant regressions, respectively (**Supplementary Material 1**). Some of the these significant regressions are shared (**Figures 5A, B**) with specific miRNAs previously cited such as miR-24 (z-score -22, p-value=1.3E-114), miR-32 (z-score -32, p-value=1.9E-287), and miR-196b and miR-23b (z-score -28.8, p-value=8.5E-183 and z-score -31, p-value=1.8E-213), the latter targeting also *MTA3*. Interestingly, miR-138 is predicted to target both *SMUG1* and *CDS1* (z-score -28, p-value=5.4E-175; z-score -30, p-value=3.4E-210). This miRNA is also actively involved in targeting lipoprotein lipase, a key lipogenic enzyme (20).

In the MEtan coding gene module we obtained 3,556 regressions, however only 431 presented a P-value <0.05 (all listed in **Supplementary Material 2**) and among these only 199 presented a z-score less than -2. Reactome analysis showed only 3 pathways with a p-value <0.05, one of these was *neutrophil degranulation*. We highlighted 2 genes associated with this pathway *Neu1* and *RhoF* associated with neutrophil activity (**Supplementary Figures 2A, B**). We identified also RNF185 a kinase involved in inflammatory processes and UGGT a glycoprotein bound to endoplasmic reticulum and downregulated in an animal model of obesity and insulin resistance (21). Two miRNAs, miR-548AQ (z-score-36, p-value=3.12E-290 and z-score-25, p-value=1E-140) and miR-6810 (z-score -7, p-value =2.3E-12 and z-score -18, p-value=8.4E-74) showed significant regressions for both genes. In addition, miR-6810 is predicted to target *RNF185* (**Supplementary Figure 3A**). Interestingly, variations of placental miR-548 methylation, and therefore, expression, were associated with insulin sensitivity in a prospective study conducted among obese mothers (22). *Neu1* and *RhoF* also showed significant regression with miR-24, already identified targeting *SMUG1* and *MTA3* in the MEgreen module. Other miRNAs already identified targeting components of the MEgreen module were miR-23b targeting *RHOF*, *MTA3*, *KDM6* and *CDS1* and miR-32 targeting *MTA3 and UGGT1*.The latter miR-196, which plays a significant role in adipogenesis, is predicted to target *RHOF* (z-score -30, p-Value= 5.4E-203). This miRNA was already identified targeting MBD4, MTA3, CDS (**Figures 4B, C**, **5A**).

MEblue, despite having a positive correlation with mLep, did not undergo mirTarRnaSeq analysis because coding genes modules negatively correlating with mLep were not identified in the WGCNA. For this reason, it was not feasible to assess potential interactions between coding genes and miRNAs for this clinical variable via mirTarRnaSeq. Similarly, since miRNAs mainly act as a transcriptional repressor, the miRNA module MEturquoise, which is negatively correlated with bwt, was not tested against coding gene modules as none were significantly positively correlated with bwt.

## Discussion

The goal of our study was to identify placental pathways and mRNA-miRNA interactions associated with the metabolic milieu among patients with obesity. Our key findings were: 1) placental expression of several components of the *Rho* network – an ubiquitous molecular signaling pathway involved in several key biological functions including inflammation - correlate with lipid metabolism pathways in the maternal-fetal dyad; 2) multiple placental genes involved in several domains of epigenetic regulation, including DNA methylation and chromatin remodeling, negatively correlate with cord blood FFA (cbFFA); 3) several miRNA-mRNA interactions are predicted among transcripts associated with cbFFA in placentas of mothers affected by obesity. While these highlighted interactions are based solely on *in silico* analysis and require functional confirmation, they are intriguing as they suggest complex relationships between placental miRNAsand endogenous mRNA with maternal and fetal lipid metabolism.

While several studies have explored placental transcriptome changes in morbidities affecting the maternal-fetal dyad, few have focused on the impact of the metabolic milieu exclusively within mothers with pre-pregnancy BMI >30. This is important for understanding how the placenta may modulate the impact of the obese environment on fetal outcomes. Recently, a correlation study (23) using WGCNA was performed by Cox et al. on a larger cohort (N = 183). They identified several genes associated with the immune and vascular systems and tissue and organ development that were correlated with both maternal pre-pregnancy BMI and newborn birthweight. It is worth mentioning that in this work samples from both overweight and obese mothers were combined. In our WGCNA analysis, conducted on 39 patients, we did not identify a group of genes correlating significantly with maternal pre-pregnancy BMI, perhaps due to the narrow BMI range (30-40) of our participants, and that all were healthy pregnancies, controlling for BMI-associated comorbidities. However, we identified 2 gene modules correlating negatively with birthweight. One, MEgray, included a heterogenous group of genes among which we identified immune and metabolic-related pathways (see **Supplementary Material 1**). The other one, MEpurple, showed overrepresentation of immune-related genes and components of the Rho family (**Table 2**; **Figure 2**).

We found significant correlations between lipid metabolism-related pathways, in both maternal (leptin) and fetal (cbFFA) compartments, with placental gene expression. Mothers with higher BMI tend to have higher lipid levels secondary to a greater adipose tissue mass (24). We found correlations with maternal leptin which is produced by adipose tissue suggesting that maternal altered lipid profile may affect placental gene expression. However, leptin is produced also by the placenta, therefore these modulations of placental transcriptomes may represent maternal adiposity or paracrine/autocrine pathways within placenta. Indeed, we identified two gene modules (MEred and ME brown) positively correlated with maternal leptin, which included several components of the Rho family. Components of the Rho family were also identified in the MEpurple module correlating with cbFFA, suggesting an important role for this family in placental crosstalk with maternal-fetal lipid metabolism. The Rho GTPase family consists of 20 proteins that play a crucial role in essential cellular processes including morphogenesis, polarity, movement, cell division and gene expression (17). Three of these genes were contained in our modules: *CDC42* and *RIF* in the MEbrown module, while *RhoB* in the MEred module, all correlating positively with maternal leptin. Most Rho GTPases cycle between an active (GDP bound conformation) and inactive (GTP bound) status, regulated by three types of proteins: Guanine nucleotide exchange factors (GEFs), GTPases-activating proteins (GAPs), and Guanine nucleotide dissociation inhibitors (GDIs) (25). Our analysis identified multiple members of this family (**Supplementary Table 2**) with different functions within the network. At the center of this complex network lies *CDC42* (**Figure 2**). Increased expression of *CDC42* is observed in livers of obese mice with the most significant changes observed at 10 weeks of age which, in human beings, correspond to the late childhood and early adulthood (26). In addition, *CDC42* also plays a significant role in glucose homeostasis and diabetes. The activation of *CDC42* has been found to be increased in CD4+ T cells in patients with obesity and asthma (27). The role of *CDC42* as regulator of the immune system and inflammation is well established as demonstrated by *CDC42* early activation following neutrophil activation and its interaction with Wiskott-Aldrich Syndrome (WAS) protein (28). WAS is a rare genetic syndrome characterized by severe immunological deficiency. Interestingly, *CDC42* and Rho family components are subject to several post-translational modifications, including association with different lipid residuals, a process called *lipidation*. Some evidence suggests that specific *lipidation* (i.e., prenylation vs palmitoylation) might determine the localization of *CDC42* within lipid membranes, leading to different downstream interaction partners and ultimately to initiation of disparate signaling cascades (29). While the potential roles of *CDC42* and the RHO GTPase family in the placenta of mothers affected by obesity need to be fully investigated, the study of CDC42 inhibition for therapeutic scope in humans has already begun. CASIN, a novel CDC42 inhibitor has been shown *in vitro* and *in vivo* to down regulate platelet activation and thrombus formation (30). It is intriguing to think that in the future novel knowledge on CDC42 and RHO GTPase function in maternal obesity could quickly translate into phase 2 experimentation.

Interestingly, cord blood free fatty acid levels (cbFFA) were correlated with the highest number of placental mRNA expression modules. This finding is particularly intriguing considering Hirschmugl et al.’s work with ^13^C-labeled FAs showing that placental lipid metabolism and storage modifies fetal delivery. (31). Lipids accumulate in the placenta and constitute an efficient and quickly available source of FA for release to the fetus. We and others have shown that lipid droplets and storage is greater in the placentas of mothers affected by obesity (7, 32). However, increased fat deposition and lipid droplet formation participate in the formation of the “lipotoxic” intrauterine microenvironment typical of obesity (7). Our analysis showed a negative correlation between cbFFA, and two genes contained in the green module, *SMUG1* and *CDS1*, both important to lipid metabolism. In rodents, SMUG1 KO, an enzyme involved in base excision repair and lipid homeostasis, has been shown to cause increased weight and fat content in one-year old mice. In addition, lipidomic profiling showed accumulation of free fatty acids and triglycerides in SMUG1 null livers (18). CDS1, an enzyme metabolically active in the inner membrane of the nuclear envelope, has been shown to participate in lipid droplet formation in yeast (33). These data suggest a possible scenario in which reduced expression of SMUG1 and/or CDS1 may predispose the placenta to fat deposition and potentially lipotoxicity.

Altogether, these data are intriguing because the overexpression of Rho components and *CDC42*, in particular, under maternal and fetal metabolic dysregulation such as caused by obesity, could be related to an increased lipid accumulation secondary to downregulation of *SMUG1* and *CDS1*. The subsequent transfer of Acyl-groups from the placental lipid droplets to Rho components and *CDC42* could enhance the inflammatory signal, or alter its functional signaling cascade, acting as the triggering link between the metabolic and inflammatory components of obesity-induced inflammation.

The identification of key components of the epigenetic machinery, whose expression in the placenta is negatively correlated with cbFFA according to the WGCNA analysis, is a novel observation of our study. The epigenome, which includes DNA methylation, histone modifications and non-coding RNA elements (including miRNAs), plays a significant role during the initial stages of development, and secondary to adverse intrauterine events such as maternal obesity (34) (List of genes in **Supplementary Table 3**). The MEgreen module contains *DNMT3A* (a methyltransferase enzyme capable of adding a novel methyl group to a cytosine base), and *MBD4* (a protein that binds methylated DNA). The levels of DNA methylation have been investigated in maternal-fetal dyads exposed to obesity. Animal studies have shown that maternal diet modulates offspring propensity to liver lipid deposition and this correlates with subtle but widespread changes in DNA methylation level (35). The same group demonstrated that enriching the diet with methyl- groups modulated methylation levels in the offspring of obese rodents (36). In humans, a global placental methylome analysis showed placental DNA methylation changes associated with pre-pregnancy BMI (37). Furthermore, this study identified similar methylation loci in placentas exposed to obesity with tissues obtained from children and adults with obesity, suggesting possible transgenerational mechanisms associated with methylation patterns. Overall, we can speculate from our data that placental expression of *DNMT3A* and *MBD4* could be the mechanism that modulates the methylome profile in the fetus following intrauterine metabolic stressors. Sub-components of chromatin remodelers involved in development (*MTA3, RSF1* and *SMARCE1)* (38) were identified in the MEgreen module. The MEgreen module also contained 17 histone genes, including variants of all the proteins that constitute the core histone of the nucleosome. All the identified components of the epigenome show a coherent functional direction towards reduced inhibition and increased gene expression. The inhibition of DNA methylation results in increased expression of genes regulated by methylation. Reduced histone production can lead to reduced nucleosome density and increased euchromatin regions in the genome (39). Finally, *MTA3* is part of the NuRD complex and *RSF1* of the SWI-Fi complex. Both complexes are inhibitory, therefore their downregulation increases gene expression (38). These findings suggest a negative modulation of the epigenome to increase gene expression in the placenta and, potentially, in the developing fetus. The extent of the impact of these effects on fetal tissues still needs to be investigated.

The study of placental miRNAs and placental target coding genes is a unique feature of this work. While miRNAs have been the focus of several studies in maternal obesity, few have examined the endogenous targets of placental miRNAs. Among all the miRNA modules analyzed, only MEturquoise, showed significant (negative) correlation with one (cbFFA) of the clinical-metabolic traits considered. Furthermore, only miR-23b, targeting *KDM6* (**Figure 5B**), was previously known to target one of coding genes considered in our analysis (40). Nevertheless, we identified several miRNAs whose altered expression has been reported in experimental models of obesity. miR-4632, targeting DNMT3, has been observed *in vitro* in hepatic cells exposed to high concentrations of palmitic acid (41).The miR-23b cluster (miR-23b-27b-24) has been shown to play an important role in NAFLD (42). miR-24, for example, which in our analysis targets SMUG1 (**Figure 5A**) and MTA3 (**Figure 5A**) are known to target *Insig*, a lipogenesis inhibitor. Overexpression of miR-24 was observed in liver of high-fat diet treated mice and its suppression prevented lipid accumulation in primary human hepatocytes (43). Likewise, miR-23, which in our analysis targets two chromatin remodeling enzymes, *MTA3* and *KDM6* (**Figures 5A, B**), and *CDS1* (**Figure 5B**) plays multifaceted roles in NAFLD pathogenesis and progression. miR-23 not only acts as an antiadipogenic regulator but can also cause lipid accumulation (44). Interestingly, these two miRNAs have been associated with the pathogenesis of cardiovascular disease (CVD) - miR-24 through the suppression of angiogenesis (45), and miR-23b contributing to atherosclerosis through promotion of inflammation and foam cell formation (46). CVD is the main cause of premature deaths among the offspring of obese mothers (5). Some miRNAs identified in our analysis have been shown to be dysregulated in other cohorts: the increased expression of miR-6845 was observed in the serum of adolescents with NASH (47), while miR-6825 in the cord blood of newborns with high adiposity (48). miR-32 predicted to target *MBD4, MTA3, SMUG1* was shown to be overexpressed in patients affected by hepatic steatosis (49).

Two miRNAs identified target enzymes involved in the process of adipogenesis. miR-138 which is predicted to target both *CDS1* and *SMUG1* (**Figures 5A, B**). Overexpression of this miRNA in primary human cells demonstrated a reduction in adipocyte formation and the maintenance of the cells in a more undifferentiated state (50). Interestingly, the target of miR-138 appeared to be lipoprotein lipase, an enzyme upregulated in obesity. Another miRNA that participates in adipogenesis identified in our analysis is miR-196. This miRNA is predicted to interact with *MBD4, MTA3, CDS1 and RHOF*. The latter is a gene we identified in the MEbrown module and is part of the Rho family. As described for miR-138, miR-196 is master regulator of adipogenesis (51). Using primary human cells, knock down of miR-196 reduces preadipocyte proliferation. Furthermore, miR-196 was associated in the same study with fat distribution (52). It is intriguing to speculate that transient exposure of the fetus and/or the newborn to miR-138 and miR-196 may block the physiological differentiation of adipocyte precursors with consequent increase of undifferentiated preadipocyte pool at birth and increased potential to accumulate fat tissue later in life. Furthermore, evidence demonstrated that committed preadipocytes are reduced in obese subjects (53).

Overall, further understanding of placental miRNA dysregulation, mRNA targets and their correlations with significant metabolic and anthropometric measures of the offspring of mothers affected by obesity is critical. The identification of specific miRNAs in the cord blood, could stratify the risk for specific morbidities associated with maternal obesity such as NAFLD and CVD, implementing preventive programs and reducing mortality and costs.

Our study presents several limitations. The study is exploratory in nature and is not powered to answer specific, hypothesis-driven questions. Furthermore, It is not possible to establish which cell population generated the molecular signals identified in our analysis. This is a relevant question, given that the expression of several components of the Rho family, for example, could originate from immune cells (macrophages or neutrophils) suggesting a reactive infiltration or from placental cells (trophoblasts) indicating possible reprogramming of these cells under metabolic pressure. A single-cell analysis may provide more specific answers to the cytological origin of the molecular signal identified. Furthermore, the preparation of cDNA libraries using poly(A) technique leads to coding gene RNA enrichment with potential loss of miRNAs. This study used as input data for the mirTarRNASeq analysis, coding and miRNA obtained from placental tissues. However, mirTarRNASeq uses Miranda databases to predict coding-miRNA interactions, therefore, in absence of dedicated functional studies, these interactions should be interpreted with caution.

Overall, this data identifies potential relationships between placental transcriptome dynamics and lipid metabolism pathways in mothers affected by obesity. While inflammation is often associated with obesity during pregnancy, the modulation of placental expression of members of the Rho network and components of the epigenome is a novel finding. Interestingly, only a limited set of placental miRNAs was found to correlate with any of the metabolic parameters analyzed (cbFFA). Our mRNA-miRNA interaction analysis identified several miRNAs-of-interest previously identified in animal models of obesity and cohorts of patients affected by obesity. Further research is necessary to determine the autocrine and/or endocrine roles of placental miRNA to impact tissue function, including fetal developing organs which may have implications for predisposing the offspring of mothers affected by obesity to cardiovascular diseases and premature death.

## Data Availability

The datasets presented in this study can be found in online repositories. The names of the repository/repositories and accession number(s) can be found below: https://doi.org/10.7910/DVN/LOIDQS.
