# Supplementary material for: Placental mRNA and miRNA dynamics associated with lipid metabolism pathways in pregnancies affected by obesity
Source: Front Endocrinol (Lausanne). 2026 Jan 28;16:1736033. doi: 10.3389/fendo.2025.1736033 (PMC12890621; doi:10.3389/fendo.2025.1736033)
Supplement: Supplementary file 4 [file DataSheet1.pdf]

frontiers

Supplementary Material

Supplementary Figures

Supp. Fig. 1 miRNA Modules-Metabolic Traits Correlations in Placentas from Mothers Affected by Obesity. Using *Whole Genome Correlations Network Analysis* (WGCNA)**,** we established correlations between placental miRNAs andmRNAs with similar patterns of expression (Modules) with significant metabolic traits of the Maternal-Fetal dyad. Each correlation shows the coefficient of correlation and (p-value). Red correlations are positive, Blue correlations are negative. Highlighted Red, miRNAs modules with significant positive correlation

cbFFA: cord blood Fatty-Acids; cblep cord blood leptin; neoadp: neonatal adiposity; bwt: birth weight; mffa: Maternal Free Fatty-Acids; mlep: Maternal Leptin; preBMI: Maternal Pre-pregnancy BMI

	prebmi	mlep	mffa	bwt	neoadp	cblep	cbFFA
MEblack	-0.38 (p=0.625)	-0.78 (p=0.221)	-0.26 (p=0.741)	-0.46 (p=0.538)	-0.45 (p=0.551)	-0.61 (p=0.385)	0.72 (p=0.279)
MEblue	0.26 (p=0.738)	0.96 (p=0.042)	0.20 (p=0.798)	0.49 (p=0.513)	0.30 (p=0.704)	0.11 (p=0.891)	-0.64 (p=0.360)
MEbrown	-0.42 (p=0.578)	-0.82 (p=0.180)	-0.40 (p=0.604)	-0.22 (p=0.782)	0.01 (p=0.990)	0.21 (p=0.794)	0.36 (p=0.636)
MEgreen	0.33 (p=0.665)	-0.84 (p=0.161)	0.44 (p=0.559)	-0.94 (p=0.061)	-0.91 (p=0.092)	-0.76 (p=0.240)	1.00 (p=0.000)
MEgrey	-0.24 (p=0.758)	0.79 (p=0.209)	-0.25 (p=0.754)	0.59 (p=0.413)	0.35 (p=0.655)	-0.15 (p=0.854)	-0.53 (p=0.469)
MEmagenta	-0.10 (p=0.902)	-0.06 (p=0.937)	-0.19 (p=0.806)	0.28 (p=0.719)	0.52 (p=0.484)	0.89 (p=0.107)	-0.39 (p=0.612)
MEred	-0.36 (p=0.644)	0.68 (p=0.320)	-0.47 (p=0.529)	0.89 (p=0.106)	0.94 (p=0.062)	0.91 (p=0.094)	-0.96 (p=0.037)
MEturquoise	0.47 (p=0.533)	-0.85 (p=0.149)	0.55 (p=0.449)	-0.98 (p=0.025)	-0.90 (p=0.103)	-0.61 (p=0.391)	0.97 (p=0.029)

\

Supp. Fig. 2 **Linear regression between selected MEtan genes and miRNAs.** Using mirTarRnaSeq we predicted linear regression between miRNAs in the MEgreen+METuquoise modules, positively correlated with cbFFA with significant genes contained in the MEtan module, negatively correlated with cbFFA Neu1 (Panel A) and RhoF (Panel B)

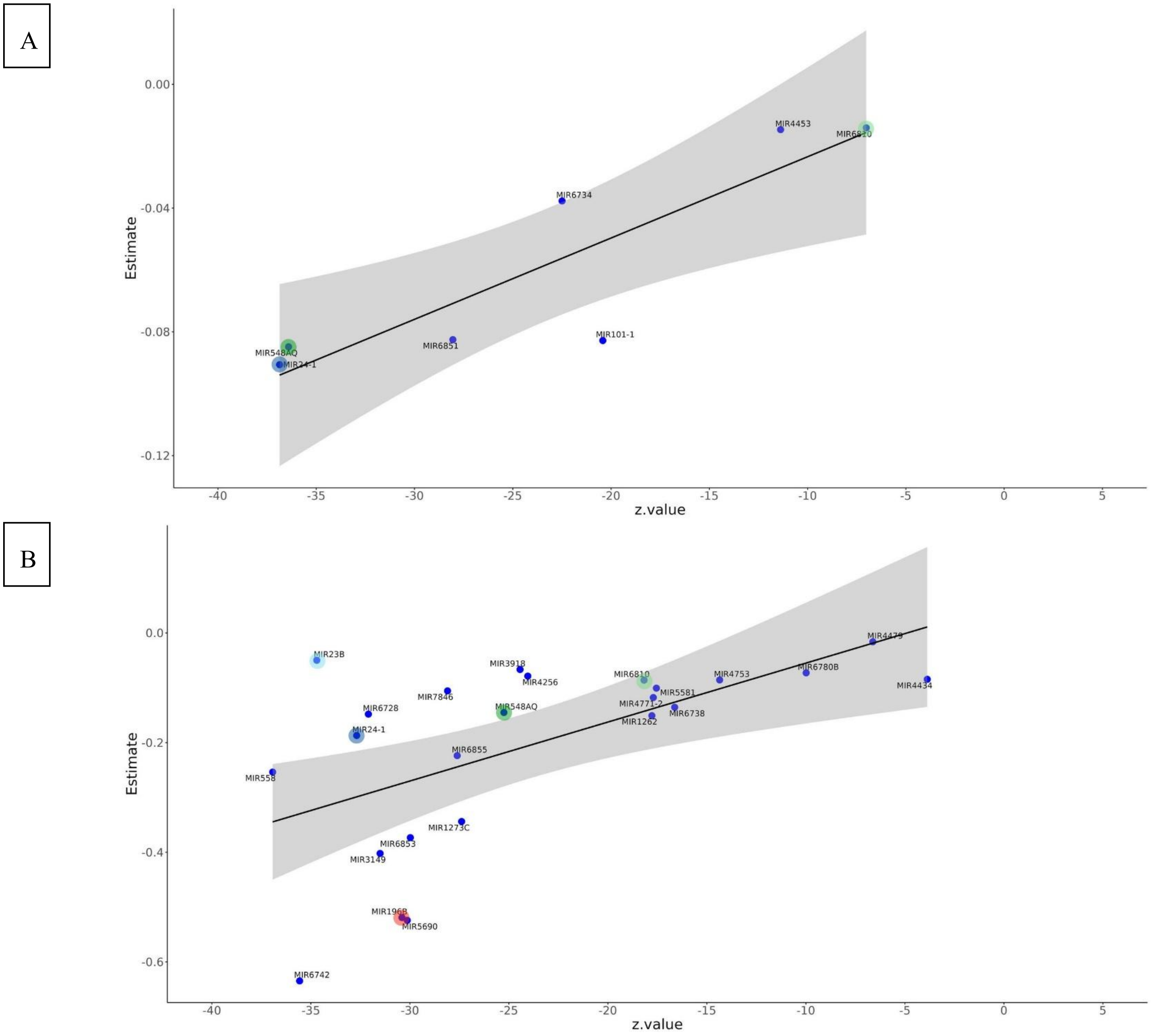

Supp. Fig.3 **Linear regression between selected MEtan genes and miRNAs.** Using mirTarRnaSeq we predicted linear regression between miRNAs in the combined MEgreen+MEturquoise modules, positively correlated with cbFFA with significant genes contained in the MEtan module, negatively correlated with cbFFA. All the miRNA-gene interactions reported have a p-value <0.05. RNF185 (Panel A) and UGGT (Panel B)

A

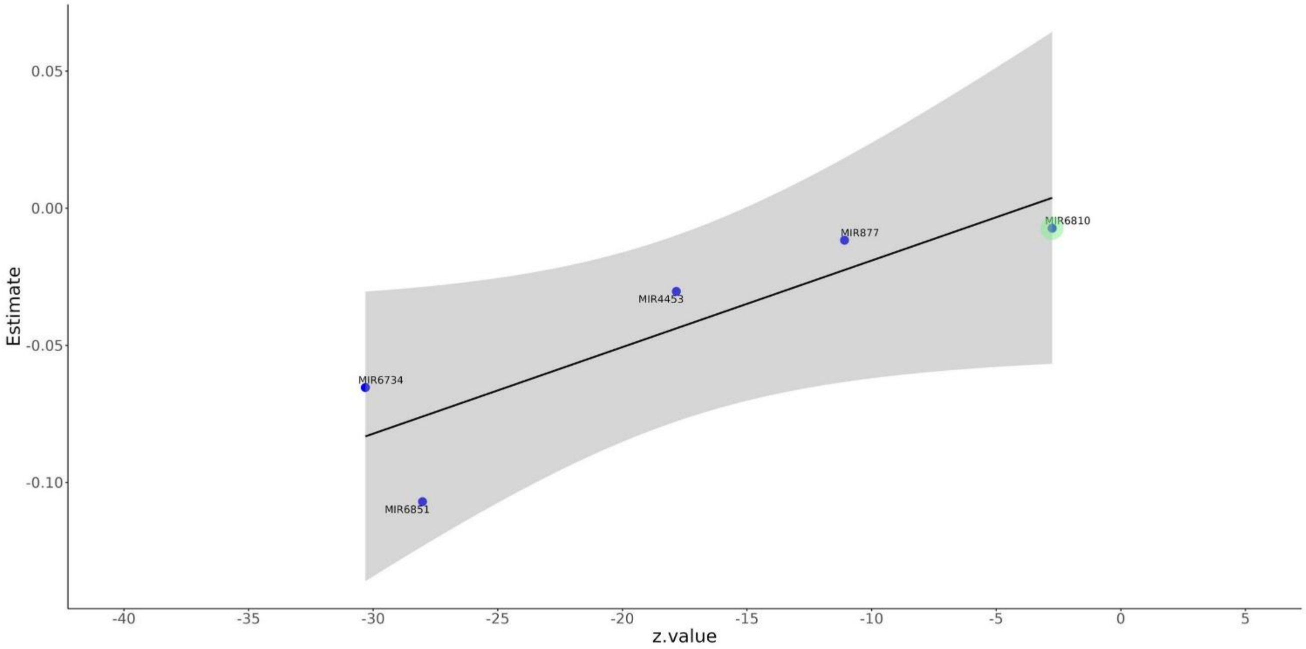

B

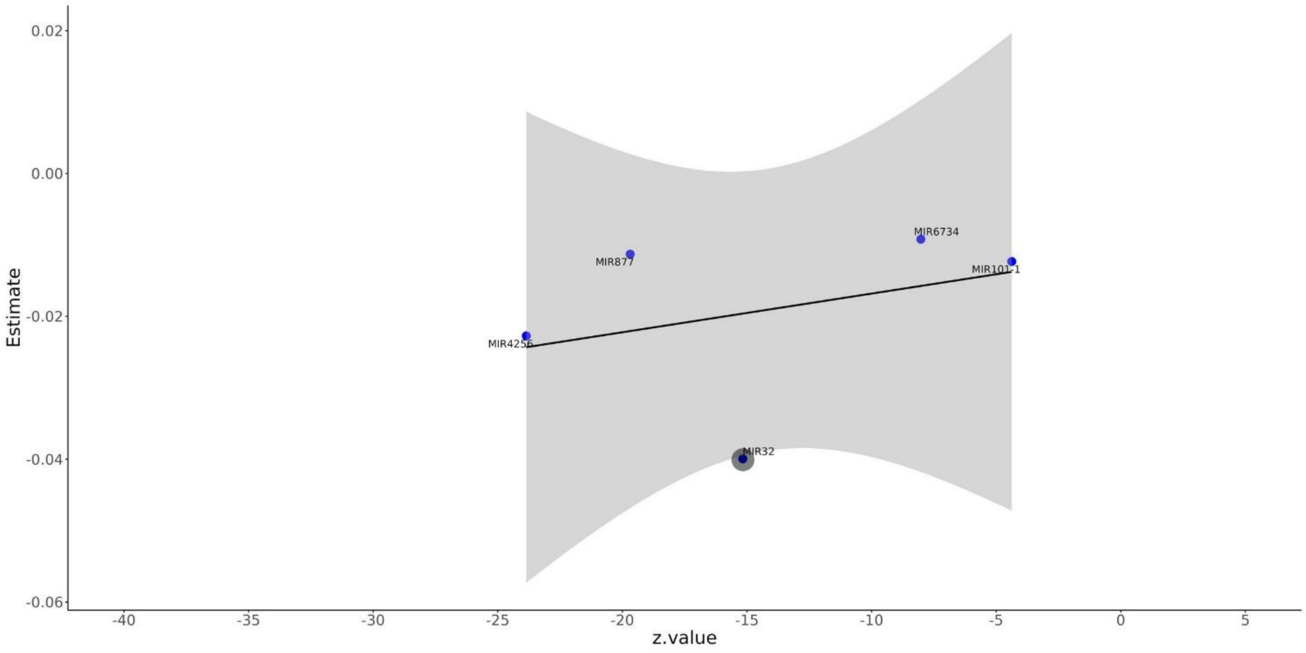

Supplementary Tables

Supp. Tab.1 Reactome Analysis of the coding-gene module "MEbrown' in placentas obtained from mothers affected by obesity. Using Reactome, an open source, peer-reviewed pathway database, we analyzed genes contained in the MEbrown module positively correlated with maternal leptin in the WGCNA analysis. The p-value and FDR reported belong to the pathway with the most significant p-value listed in that specific system. The pathways reported are all the pathways that presented p-value <0.05 in the Reactome analysis for all the genes listed in the MEbrown module.

frontiers

System	Pathway	Significant Genes	P-value	FDR
Immune System	Antigen processing: Ubiquitination and Proteasome degradation	UBE3A-4A-3C, UBA5-6, UBE2G2, FBXl4, CUL3-5	0.0001	0.17
Cell Cycle	Cohesin Loading onto Chromatin; Establishment of Sister Chromatid Cohesion; E2F-enabled inhibition of pre-replication complex formation; Nuclear Pore complex	STAG1-2-3-RAD21, PDS5A-B, NUP50-58-88-133-153-155	0.00015	0.17
Disease	ALK mutants bind TKIs; Defective TPR; Loss of Function of SMAd4 in Cancer, SMAd4 MH2 Domain Mutants in Cancer; XAV939	CLTC, EIF2AK3, EML4	0.00026	0.19
Metabolism	Regulation of Glucokinase-by-Glucokinase regulatory protein	NUP50-58-88-133-153-155	0.029	0.99
Other		CDC42, CDC42ES1, FGD4, FARP2, DOCK8-11		

Supp. Tab.2 Components of the RHO network included in the Mebrown, MEred and MEpurple modules. *CDC42, RIF* and *RHOb* are part of the RHO-GTPase family which includes 20 members. The remaining genes are part of a network that facilitates and modulates RHO family activities. GEF: Guanidine Nucleotide Exchange; GAP: GPAses activating Proteins

**Module**	RHO GTPase	GEF	GAP/Inhibitor	Effectors
MEbrown	*CDC42, RIF*	*Vav3, FGD4, FARP2 DOCK1-8-11*		
MEred	*RHOB*		*ARHGAP17-30-21*	*PAK4,ROCK1,* CDC42PB, CDCEP4
MEpurple		*ARHGEF10-25 CDC42EP3-4-5*	*ARHGAP1-33*	*PAK2, WASF, CDC42EP1-2-5*

Supp. Tab.3 Genes in the MEgreen listed in Reactome pathways. These are genes, contained in the MEgreen module, which constitute pathways with significant p-value (<0.05) in the Reactome analysis (36 genes in total). Notable for significant overrepresentation of genes associated with the Epigenetic machinery (24 genes). Also, genes associated with lipid metabolism.

Biological Function		Genes
Epigenome	(#19)	Histones and Histone modifier<br>-H1-2, H2AC6-7-11, H2BC4-5-6-8-12-21, H3C4-12, H4C5-8-9-14-15<br>-KDM6A
	(#2)	DNA Methylation<br>-DNMT3<br>-MBD4
	(#3)	Chromatin Remodelers<br>-MTA3<br>-RSF1<br>-SMARCE1
Lipid Metabolism	(#4)	-SMUG1, CDS1, YWHAZ,PIAS4
Others	(#8)	TDG, CAST, CCNH, KPNB1, Top3A, AMFR, SEC13, RAE1

Supp. Tab.4 Reactome Analysis of the coding-gene module "MEtan' in placentas obtained from mothers affected by obesity. Using Reactome, an open source, peer-reviewed pathway database, we analyzed genes contained in the MEtan module positively correlated with maternal leptin in the WGCNA analysis. The p-value and FDR reported belong to the pathway with the most

significant p-value listed in that specific system. The pathways reported are all the pathways that presented p-value <0.05 in the Reactome

System	Pathway	Significant Genes	P-value	FDR
Metabolism of Protein	Asparaginase N-linked glycosylation; Calnexin/Calreticulin cycle; SRP-dependent cotranslational protein target to membrane; ER quality control Compartment; N-Glycan trimming in the ER and Calnexin/Calreticulin Cycle;	UGGT1, RNF185, DDOST	0.001.35	0.88
Immune System	Neutrophil Degranulation	Neu1, RHOF, RPN1-2	0.002.4	0.88
Signal Transduction	NOTCH2 activation and Transmission of Signal to Nucleus; MET activates RAP1 and RAC1; RHOF GTPase; RND1 GTPase cycle; TGFBR3 PTM regulation	ALG3, NOTCH2	0.000002.2	0.0012
Disease	Defective PGM1 causes PGM-CDG; Defective HK1 causes hexokinase deficiency; Defective DPAGT1 causes CDG-1j; Defective ALG3 causes CDG-1d;	ALG3	0.00001	0.0025
Gene Expression	Runx2 regulates genes involve din cell migration	ITGA5	0.04.6	0.88

analysis for all the genes listed in the MEred mod
